# Extranodal involvement defines distinct immune-molecular phenotypes and clinical outcomes in diffuse large b-cell lymphoma

**DOI:** 10.1007/s10238-026-02268-3

**Published:** 2026-07-26

**Authors:** Yiliya Ahongjiang, Lihua Qiu, Min Li, Biwen Sun, Huilai Zhang, Chen Tian

**Affiliations:** Key Laboratory of Cancer Prevention and Therapy, Tianjin; Tianjin’s Clinical Research Center for Cancer, Tianjin Medical University Cancer Institute and Hospital, National Clinical Research Center for Cancer, Tianjin, 300060 People’s Republic of China

**Keywords:** Diffuse large B-cell lymphoma, Extranodal involvement, Prognosis, Tumor microenvironment, RNA sequencing, Targeted sequencing, Immune heterogeneity

## Abstract

Extranodal involvement (ENI) is incorporated into routine risk assessment for diffuse large B-cell lymphoma (DLBCL), but treating ENI as a single binary adverse feature may conceal clinically relevant heterogeneity in extranodal burden, anatomical distribution, treatment feasibility, and immune-molecular context. We evaluated ENI as a burden- and site-aware phenotype rather than as a uniform descriptor. We retrospectively studied 710 adults with newly diagnosed DLBCL treated with first-line immunochemotherapy between June 2011 and December 2024. Outcomes were analyzed according to ENI status, number of extranodal sites, and involved organs. Targeted sequencing was available in 176 tumors, and RNA sequencing was performed in 107 quality-controlled tumor samples. Clinical models were interpreted as adjustment models because ENI burden overlaps with established risk factors; site-specific and molecular analyses were prespecified as exploratory and were interpreted with attention to available-case denominators, treatment heterogeneity, sparse subgroups, and multiplicity. ENI was associated with inferior overall survival (OS) and progression-free survival (PFS) in unadjusted analyses. Multisite ENI was enriched for adverse baseline features, including advanced Ann Arbor stage, elevated lactate dehydrogenase (LDH), higher International Prognostic Index (IPI), and impaired performance status. After multivariable adjustment, the independent prognostic contribution of ENI burden was attenuated, indicating substantial clinical overlap with systemic disease burden and host fitness. Several anatomical sites showed candidate adverse signals, but rare-site estimates were limited by small subgroup sizes, wide confidence intervals, and multiple testing. Targeted sequencing and RNA-seq suggested heterogeneous genomic and immune-transcriptional patterns across ENI categories; these results should be regarded as hypothesis-generating because of tissue availability, tumor-only sequencing in many cases, modest molecular sample sizes, and lack of orthogonal immune validation. ENI in DLBCL is best interpreted as a clinically heterogeneous disease descriptor rather than a single uniform prognostic category. ENI burden and anatomical distribution provide useful descriptive and prognostic context, but they should be interpreted alongside established clinical risk, treatment intensity, censoring patterns, tissue-sampling constraints, and molecular ascertainment limitations. Exploratory genomic and immune-transcriptional correlates identified in this study require prospective multicenter validation with harmonized staging, treatment-intensity annotation, matched-normal sequencing, and spatial or cellular immune profiling before ENI-informed biological or therapeutic stratification can be applied clinically.

**Graphical abstract:**

**Figure:**
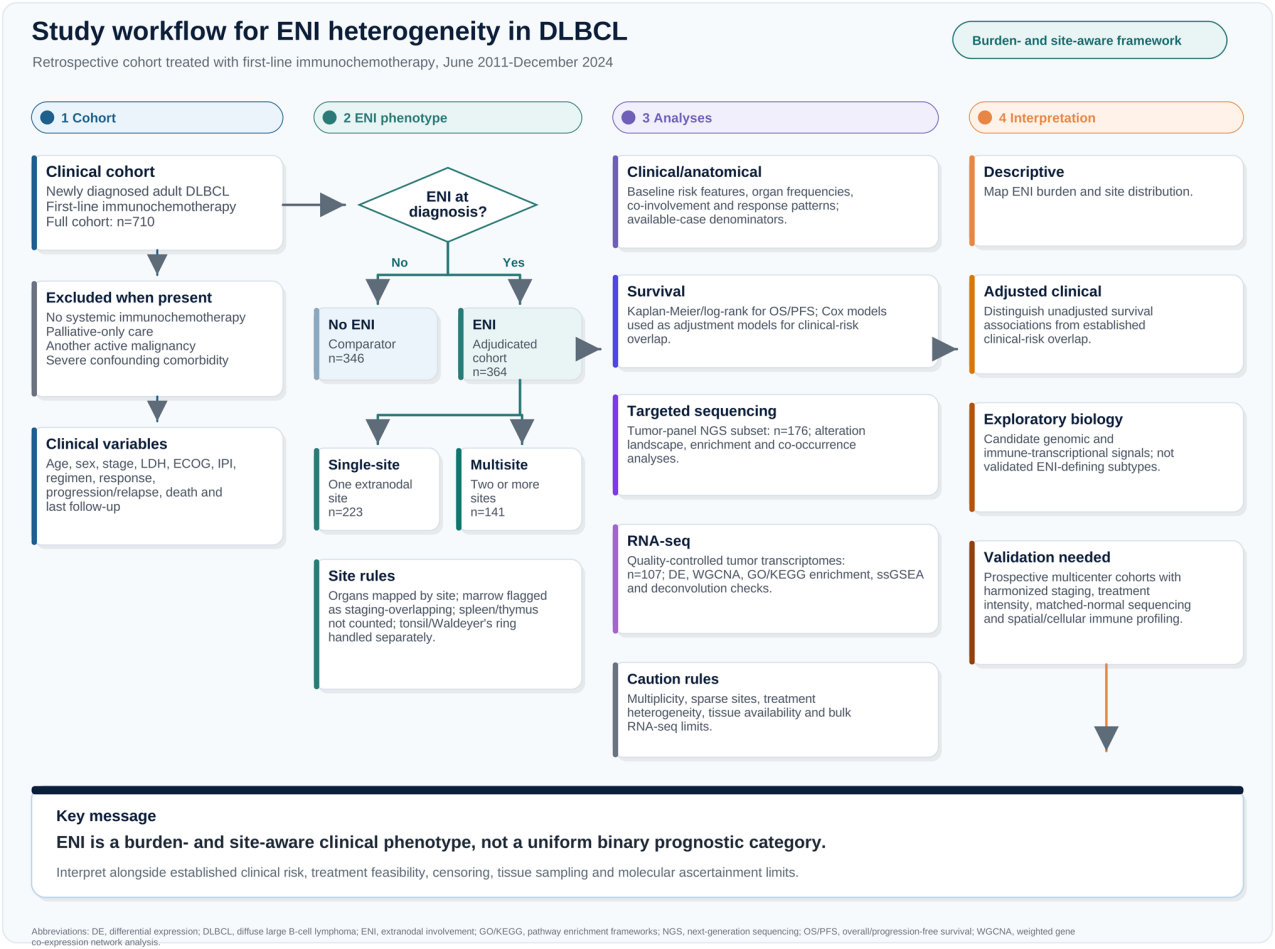

Study design, ENI boundary definitions, statistical safeguards, and multi-omics integration

**Supplementary Information:**

The online version contains supplementary material available at 10.1007/s10238-026-02268-3.

## Introduction

Diffuse large B-cell lymphoma (DLBCL) remains clinically heterogeneous despite advances in diagnostic classification, immunochemotherapy, and molecular taxonomy [1, 2]. Gene-expression profiling established the cell-of-origin framework, and subsequent genomic studies have divided DLBCL into molecular groups with distinct pathogenic programs and therapeutic vulnerabilities [3–8]. In routine clinical practice, however, risk estimation still relies heavily on readily available clinical variables, including age, stage, lactate dehydrogenase (LDH), Eastern Cooperative Oncology Group (ECOG) performance status, and extranodal involvement (ENI) [9, 10].

ENI is common at presentation and is included in major prognostic systems, including the International Prognostic Index (IPI) and the NCCN-IPI [9, 10]. Its practical value lies in its apparent simplicity, but this simplicity can be misleading. Extranodal disease may reflect localized organ involvement, disseminated tumor burden, organ-specific tropism, differences in biopsy and imaging practices, or treatment constraints imposed by patient fitness and anatomical risk. Therefore, binary ENI classification may obscure clinically relevant differences related to the number of involved organs, the specific anatomical sites affected, and the overlap between ENI and established risk factors.

The biological basis of this non-uniformity remains incompletely defined. Extranodal spread may arise from systemic dissemination, organ-specific homing, permissive stromal niches, immune escape, or combinations of these mechanisms. In DLBCL, disruption of antigen presentation and immune recognition, including alterations involving B2M and HLA class I, is a recognized route of immune evasion [11, 12]. Transcriptomic studies have also shown that tumor microenvironmental programs contribute to DLBCL biology and outcome [13, 14]. Whether immune-molecular features differ reproducibly by ENI burden or organ pattern remains uncertain, particularly when molecular sampling is constrained by real-world tissue availability.

We therefore integrated clinical, anatomical, targeted genomic, and transcriptomic data in a retrospective cohort of newly diagnosed DLBCL. The primary objective was not to replace established prognostic models, but to determine whether ENI contains additional clinically interpretable structure when analyzed by burden and anatomical distribution. A secondary exploratory objective was to identify genomic and immune-transcriptional patterns that may generate testable hypotheses for future prospective studies. Throughout the manuscript, we distinguish descriptive, adjusted clinical, and exploratory molecular findings to avoid overinterpretation of retrospective and sparse-subgroup signals.

## Methods

### Study design and patient cohort

This retrospective cohort study included adults aged 18 years or older with newly diagnosed DLBCL who received first-line immunochemotherapy at our institution between June 2011 and December 2024. Each case was diagnosed as DLBCL on formal pathological evaluation of diagnostic biopsy specimens by institutional pathologists, integrating histomorphologic assessment with immunophenotypic findings documented in the pathology report. Eligible cases were defined and harmonized according to the World Health Organization classification of lymphoid neoplasms and included DLBCL, not otherwise specified, as well as recognized DLBCL-related subtypes when applicable [1]. The study was designed to evaluate ENI burden and anatomical distribution in relation to clinical outcome and exploratory molecular features.

After application of prespecified eligibility criteria, 710 patients were included in the clinical cohort. Of these, 364 had ENI at diagnosis and were evaluable for extranodal burden, anatomical distribution, treatment response, and survival analyses when the corresponding variables were available. Patients were excluded if they did not receive systemic immunochemotherapy, received only palliative care, had another active malignancy, or had severe comorbid illness expected to confound assessment of lymphoma-directed therapy. Patient screening and exclusion are summarized in Supplementary Figure S1.

All analyses used predefined but not fully identical analysis sets. The full clinical cohort comprised 710 patients. The primary ENI-burden survival cohort comprised 364 patients with adjudicated extranodal involvement and final classification as single-site or multisite ENI. Baseline, anatomical, response, survival, genomic, and transcriptomic analyses used variable-specific available-case denominators because retrospective annotations were not uniformly complete. Denominators may therefore differ across tables and figures according to the analytic purpose and the required level of annotation completeness. Apparent differences between the primary ENI-burden cohort size and selected table-level denominators should be interpreted as available-case reporting rather than as changes in the underlying clinical cohort.

Clinical information was abstracted from electronic medical records using a standardized data-collection protocol. Recorded variables included age, sex, Ann Arbor stage, LDH, ECOG performance status, IPI, cell-of-origin subtype when available, first-line regimen, response assessment, progression or relapse date, death date, and last follow-up date. The study was conducted in accordance with the Declaration of Helsinki, approved by the institutional ethics committee, and performed with written informed consent according to institutional requirements.

Cell-of-origin subtype was recorded when available as part of routine pathology workup. In contrast, detailed morphologic subtype annotation, such as centroblastic, immunoblastic, or anaplastic morphology, was not uniformly archived across the retrospective cohort and therefore could not be robustly correlated with genomic findings in the full dataset; we now state this explicitly and regard it as an important priority for future prospective studies.

### Definition and classification of extranodal involvement

ENI was defined as lymphoma infiltration involving organs or tissues outside conventional nodal structures. Boundary organs were handled conservatively to reduce classification-driven bias. Spleen and thymus were not counted as extranodal sites in the primary burden definition because of their overlap with lymphoid-organ staging. Biopsy-confirmed bone marrow involvement was recorded as a distinct anatomical site for burden mapping but was flagged a priori as a staging-overlapping variable because marrow disease contributes directly to Ann Arbor stage IV and may correlate with IPI-related risk. Accordingly, analyses involving ENI number were interpreted together with stage, LDH, ECOG performance status, IPI-related covariates, and sensitivity definitions excluding marrow from the extranodal-site count when raw annotations allowed comparison.

Patients with ENI were categorized by the number of involved extranodal anatomical sites. Single-site ENI referred to one involved non-nodal anatomical site, whereas multisite ENI referred to two or more such sites. Recorded anatomical categories included stomach, intestine, bone, bone marrow, lung, central nervous system (CNS), liver, pancreas, kidney/adrenal gland, nasal cavity, thyroid, breast, uterus/ovary, testis, mediastinum, and skin. Lesions were identified through clinical examination, cross-sectional imaging, histopathology, bone marrow assessment, cerebrospinal fluid evaluation when clinically indicated, and multidisciplinary review of the diagnostic record. The mediastinal category was retained only when records supported extranodal mediastinal or soft-tissue involvement rather than conventional nodal mediastinal disease; given its rarity and potential anatomical ambiguity, it was treated as an exploratory sparse-site variable. Organ-level analyses had two distinct purposes: organ frequencies were described in the full clinical cohort to provide an anatomical map, whereas organ-defined variables were compared between single-site and multisite ENI within the burden-classified ENI subset. Because these analyses used different denominators and different annotation requirements, organ counts in descriptive figures and burden-comparison tables are not expected to be numerically identical.

Waldeyer’s ring and tonsillar involvement were handled as a prespecified boundary category rather than as unqualified extranodal disease. Although tonsillar disease was retained in the anatomical inventory for comparability with head and neck lymphoma literature, Waldeyer’s ring occupies a recognized borderline position between mucosa-associated lymphoid tissue and conventional lymphatic tissue. Tonsillar findings were therefore reported separately and were not used alone for mechanistic inference. Where feasible, sensitivity analyses treating Waldeyer’s ring separately from strict extranodal organs were used to assess whether principal ENI-burden results depended on this classification choice.

### Treatment, response assessment, and follow-up

First-line immunochemotherapy was selected according to institutional practice, disease characteristics, and patient fitness. Rituximab plus cyclophosphamide, doxorubicin, vincristine, and prednisone (R-CHOP) was the standard backbone [15]. Dose-adjusted R-CHOP was considered R-CHOP-based for survival analyses. Patients unsuitable for anthracycline-based therapy received alternative regimens, including etoposide-containing approaches such as R-CEOP, at the discretion of the treating physicians. Patients with CNS involvement received CNS-directed therapy, including high-dose methotrexate-based treatment when clinically appropriate. Treatment intensity was classified at minimum as standard anthracycline-containing immunochemotherapy versus reduced-intensity or anthracycline-sparing treatment. When cycle-level dosing data were retrievable, relative dose intensity (RDI) was incorporated as a sensitivity covariate; when complete RDI reconstruction was not possible across the 2011–2024 treatment window, treatment-intensity class was retained as the more reproducible adjustment variable and incomplete RDI ascertainment was treated as a limitation.

Treatment heterogeneity was considered a major potential confounder rather than a minor technical issue. Although all patients received first-line immunochemotherapy, regimen selection, dose intensity, CNS-directed therapy, treatment interruption, and tolerance may have differed according to age, performance status, systemic disease burden, and anatomical involvement. Consequently, survival differences associated with ENI patterns were interpreted as clinical associations within a treated cohort, not as purely disease-intrinsic biological effects.

Treatment response was assessed using the Lugano classification and categorized as complete response, partial response, stable disease, or progressive disease [16]. Response analyses were restricted to patients with documented post-treatment assessment sufficient for formal Lugano categorization. Patients without evaluable response data were excluded from response-specific tabulations but retained in survival analyses when follow-up data were available. Missing response information reflected incomplete post-treatment imaging documentation, treatment interruption before formal response assessment, transfer of care, loss to response follow-up, or insufficient documentation to assign a response category. Therefore, response analyses were interpreted as descriptive available-case analyses rather than unbiased comparative efficacy estimates.

Patients were followed from the start of first-line treatment until death or last available follow-up. Overall survival (OS) was defined as time from treatment initiation to death from any cause or last follow-up. Progression-free survival (PFS) was defined as time from treatment initiation to progression, relapse, death from any cause, or last follow-up, whichever occurred first. Because accrual extended to December 2024, long-term estimates were interpreted with particular attention to censoring, numbers at risk, and follow-up maturity at later time points.

### Targeted next-generation sequencing and variant analysis

Targeted next-generation sequencing was performed in 176 patients with diagnostic tumor tissue of sufficient quality. Tumor specimens were obtained from nodal or extranodal biopsies and processed as formalin-fixed paraffin-embedded tissue using standard laboratory procedures. Genomic DNA was extracted according to institutional protocols, and DNA quantity and quality were assessed before library preparation. Tissue availability was not random; consequently, molecular analyses were considered exploratory and potentially subject to selection bias related to biopsy site, tumor content, tissue preservation, and clinical workflow.

Sequencing used a lymphoma-focused custom gene panel. Library preparation included DNA fragmentation, end repair, adapter ligation, amplification, hybridization capture, and enrichment, followed by paired-end sequencing on an Illumina platform. Raw reads were assessed using FastQC [17], trimmed using Trimmomatic [18], and aligned to hg19/GRCh37 using BWA-MEM [19]. Alignment files were sorted, indexed, duplicate-marked, and processed using SAMtools and related tools [20]. Panel design, target-region selection, coverage assessment, and variant filtering followed the institutional sequencing workflow used for the corresponding clinical sequencing period. Because complete panel-design files and run-level coverage summaries were not uniformly available for inclusion in the manuscript package, the genomic results are presented as exploratory tumor-panel alteration patterns, with emphasis on transparent interpretation of tissue availability, tumor-only sequencing, and retrospective technical metadata limitations.

Single-nucleotide variants and insertions/deletions were identified using a standardized workflow consistent with GATK best-practice principles [21]. Variants were annotated with the Ensembl Variant Effect Predictor and public reference databases [22]. Coding alterations were classified by predicted consequence, including missense, nonsense, frameshift, splice-site, and other protein-altering events. Because sequencing was performed in a tissue-available subset and matched-normal sequencing was not uniformly available, genomic findings are reported as tumor-panel alteration patterns rather than definitive somatic driver events. Variant filtering considered variant allele fraction, read depth, strand balance, population-frequency annotation, predicted coding consequence, and recurrent technical artifacts according to the institutional workflow used at the time of testing. The absence of uniformly retrievable matched-normal data, run-level coverage summaries, and complete filtering-threshold records across the full retrospective sequencing interval is acknowledged as a limitation of the exploratory genomic analysis.

Mutation frequencies were summarized across the sequenced cohort and compared between single-site and multisite ENI. Enrichment analyses used odds ratios with appropriate statistical testing and multiplicity adjustment where supported by the analysis framework. Pairwise co-occurrence and mutual-exclusivity patterns were explored separately in single-site and multisite ENI groups. These analyses are sensitive to mutation burden, panel design, filtering choices, tumor-only inference, biopsy-site selection, and sample size; therefore, they were not used to define molecular subtypes or to infer causal dependencies.

### RNA sequencing, transcriptomic processing, and quality control

RNA-seq analysis was performed in 107 patients whose tumor samples passed transcriptomic quality control. For single-site ENI, tissue was obtained from the involved lesion when available. For multisite ENI, the analyzed specimen was selected according to tissue availability, diagnostic adequacy, and tumor content rather than systematic sampling of all involved organs. This pragmatic sampling strategy reflects real-world tissue availability but may not capture spatial variation across lesions, organs, or immune microenvironments.

Sequencing reads were processed through a uniform computational workflow. Reads were aligned to the human reference genome, and gene-level count matrices were generated using consistent parameters. Genes with consistently low expression were removed, and expression values were normalized and log-transformed before downstream analysis. Sample-level quality was evaluated by sequencing metrics and unsupervised clustering; samples with inadequate library quality, poor alignment, low tumor signal, or outlier global-expression patterns were excluded. Quality-control metrics included read depth, Q30, mapping rate, duplication rate, RNA integrity or equivalent FFPE quality metrics, and prespecified exclusion thresholds for the 107 retained transcriptomes.

### Differential expression, WGCNA, functional enrichment, and immune-signature analysis

Differential expression analysis compared single-site and multisite ENI transcriptomes. Statistical modeling used empirical variance moderation implemented in established RNA-seq and microarray analysis frameworks [23], with count-specific mean–variance modeling applied when appropriate. Differentially expressed genes were defined by an absolute log2 fold-change of at least 1 and an FDR-adjusted q value below 0.05. Where sample size permitted, sensitivity models considered clinically relevant covariates, including stage, LDH, tissue site, treatment era, and treatment intensity. Because residual confounding and tissue-site imbalance could not be fully eliminated, transcriptomic results were interpreted as exploratory biological signals.

Weighted gene co-expression network analysis (WGCNA) was applied to normalized expression data to identify co-expressed gene modules [24]. Candidate soft-thresholding powers were evaluated to approximate scale-free topology, and modules were identified using hierarchical clustering and dynamic tree cutting. Module eigengenes were correlated with clinical phenotypes, including ENI pattern. WGCNA results were interpreted as exploratory module-level associations because full tuning parameters, outlier-removal rules, and module-preservation diagnostics were not uniformly available for the retrospective manuscript package.

Genes overlapping between differentially expressed genes and ENI-associated WGCNA modules were carried forward for Gene Ontology and Kyoto Encyclopedia of Genes and Genomes enrichment analyses using established tools [25]. Enrichment results were considered significant after FDR correction. Immune-cell-related transcriptional patterns were estimated from bulk RNA-seq using single-sample gene-set enrichment analysis in the GSVA framework [26]. To reduce dependence on a single transcriptional proxy, ssGSEA results were cross-checked, when input data quality and normalization were adequate, using deconvolution approaches such as CIBERSORTx and/or xCell [27, 28]. Analytical resources and immune-signature sources explicitly cited in the manuscript are summarized in Supplementary Table S2. Because bulk RNA-seq cannot directly quantify immune-cell abundance, localization, or functional state, all immune-infiltration results were interpreted as relative transcriptional estimates rather than direct cellular measurements.

WGCNA and enrichment analyses were used to prioritize co-expression modules and pathway-level hypotheses. Given the modest RNA-seq sample size, the exploratory nature of module detection, and the lack of orthogonal immune profiling in the current dataset, module-trait associations were interpreted as supportive biological context rather than independently validated molecular subtypes.

### Statistical analysis

Categorical variables were summarized as counts and percentages and compared using Pearson chi-square or Fisher exact tests, as appropriate; Fisher exact testing was preferred when expected cell counts were small. Continuous variables were summarized using medians with ranges or interquartile ranges and compared using the Mann–Whitney U test. Percentages were calculated using the evaluable denominator for each variable, and missingness was not assumed to be random.

Survival outcomes were estimated using the Kaplan–Meier method and compared using log-rank tests. Multivariable Cox models evaluated whether ENI-related variables retained prognostic information in the context of established clinical risk factors. Given expected collinearity among stage, LDH, ECOG performance status, IPI, ENI burden, and selected anatomical sites, these models were interpreted as adjustment models rather than definitive causal or automated variable-selection models. Variables were selected to balance clinical relevance and statistical stability, and results were interpreted cautiously when conceptually overlapping covariates were included in the same model. Hazard ratios were reported with 95% confidence intervals. Proportional-hazards assumptions were evaluated using Schoenfeld residuals or equivalent diagnostics; when violations were detected, time-varying coefficients or stratified analyses were considered. Model discrimination was summarized using Harrell’s C-index where appropriate.

Multiplicity control was aligned with the purpose of each analysis. Differential expression, pathway enrichment, immune-signature comparisons, and mutation-enrichment analyses were evaluated using FDR-adjusted q values when supported by the corresponding framework. For anatomical site screening and rare-site survival analyses, nominal p values were presented to describe exploratory signals, whereas multiplicity, subgroup size, confidence-interval width, event numbers, and biological plausibility guided interpretation. Findings not supported by FDR-adjusted robustness were described as exploratory and hypothesis-generating rather than definitive. For rare anatomical subgroups with very small event counts, including mediastinal involvement, conventional maximum-likelihood Cox estimates were considered potentially unstable and were interpreted descriptively. Clinical and survival analyses were conducted using SPSS version 26.0 and R version 4.3.3. Transcriptomic and bioinformatic analyses were performed in R using established Bioconductor and CRAN packages, including limma, WGCNA, clusterProfiler, GSVA, maftools, and survival-modeling packages where applicable [23–26, 29, 30]. Key analytical resources explicitly cited in the manuscript are summarized in Supplementary Table S2.

### Sensitivity and interpretation hierarchy

To preserve interpretability without changing the reported figure or table content, analyses were organized according to a predefined hierarchy. First, descriptive analyses mapped ENI burden, anatomical distribution, and response patterns using available-case denominators. Second, survival analyses separated unadjusted Kaplan–Meier comparisons from multivariable Cox adjustment models. Third, rare anatomical sites and molecular comparisons were interpreted only as exploratory signals when subgroup size, event count, or multiplicity limited inference. Finally, genomic and immune-transcriptional results were not used to redefine clinical risk groups, because they were derived from tissue-available subsets and require external validation.

## Results

### Clinical features associated with extranodal disease burden

**Fig. 1:**
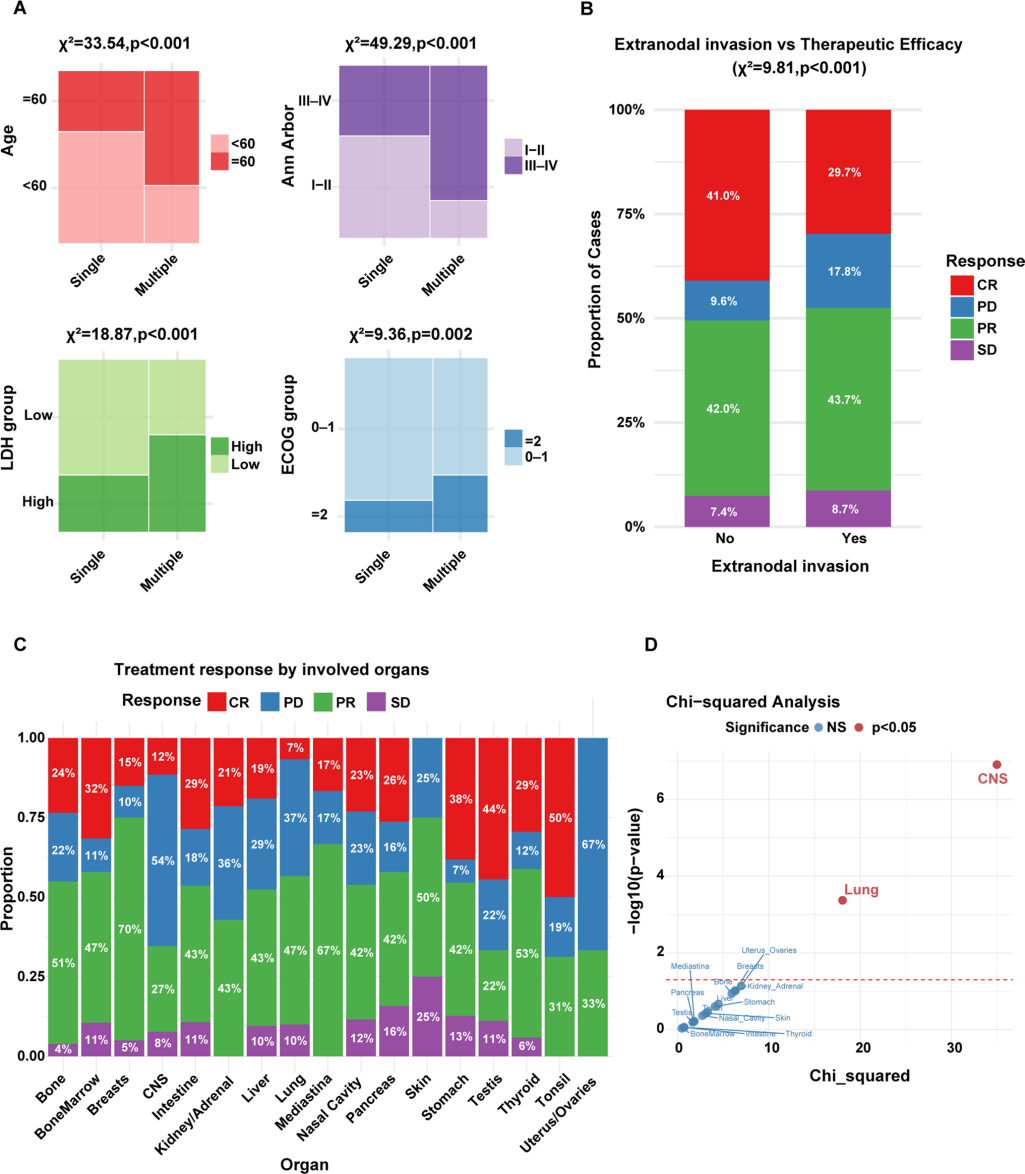
Clinical correlates of ENI burden and organ-specific treatment response in DLBCL. (**A**) Baseline clinical features were compared between single-site and multisite ENI among ENI patients (n = 364) using chi-square or Fisher exact tests as appropriate. Multisite ENI was associated with age >  = 60 years, Ann Arbor stage III-IV, elevated LDH, and ECOG performance status >  = 2. (**B**) Treatment-response distribution was compared between patients with and without ENI in the full clinical cohort using available response data. This comparison suggested a nominal difference, with lower complete-response and higher progressive-disease proportions among ENI patients. (**C**) Response distribution by involved extranodal organ. (**D**) Organ-response screening showed nominal associations for CNS and lung involvement. Response analyses are descriptive available-case analyses and were not adjusted for stage, LDH, ECOG status, treatment regimen, treatment intensity, or disease burden. CR, complete response; DLBCL, diffuse large B-cell lymphoma; ECOG, Eastern Cooperative Oncology Group; ENI, extranodal involvement; LDH, lactate dehydrogenase; PD, progressive disease; PR, partial response; SD, stable disease

The clinical cohort included 710 newly diagnosed patients with DLBCL who received first-line immunochemotherapy. ENI was identified at diagnosis in 364 patients in the primary burden-classified clinical cohort, including 223 patients with single-site ENI and 141 patients with multisite ENI for survival analyses. Baseline clinical variables were evaluated according to ENI burden (Fig. 1A) and summarized in Table 1. Within the primary ENI cohort, the median age was 58 years (range, 19–82). Sex information was evaluable in 363 patients, including 192 males and 171 females. Because baseline, anatomical, response, survival, genomic, and transcriptomic annotations were not uniformly complete, variable-specific available-case denominators were used throughout the Results.

**Table 1:** Associations between ENI number and clinical or anatomical factors in DLBCL. Variables are compared between single-site and multisite ENI using Pearson chi-square or Fisher exact tests as appropriate. Counts and percentages are shown for each category, and percentages were calculated using the available denominator for each variable. Because baseline, anatomical, response, and burden-classification variables were not uniformly complete in the retrospective dataset, the number of evaluable cases may differ across variables. The primary burden-classified ENI cohort used for survival analyses comprised 364 patients, whereas Table 1 presents variable-specific available-case tabulations. P values are descriptive and were not intended to define independent predictors. Rare organ-defined comparisons, staging-overlapping variables such as bone marrow involvement, and boundary categories such as tonsil/Waldeyer’s ring should be interpreted cautiously. Abbreviations: DLBCL, diffuse large B-cell lymphoma; ECOG, Eastern Cooperative Oncology Group; ENI, extranodal involvement; IPI, International Prognostic Index; LDH, lactate dehydrogenase

Characteristic	Cases	Single	Multiple	P value
Age				
< 60	192	145 (75.52%)	47 (24.48%)	p < 0.001
> = 60	172	78 (45.35%)	94 (54.65%)	p < 0.001
Sex				
Female	171	110 (64.33%)	61 (35.67%)	0.288
Male	192	112 (58.33%)	80 (41.67%)	0.288
Elevated serum LDH				
High	166	85 (51.20%)	81 (48.80%)	p < 0.001
Low	197	138 (70.05%)	59 (29.95%)	p < 0.001
Subtype				
GCB	145	96 (66.21%)	49 (33.79%)	0.128
non-GCB	173	99 (57.23%)	74 (42.77%)	0.128
Ann Arbor stage				
I-II	163	133 (81.60%)	30 (18.40%)	p < 0.001
III-IV	202	91 (45.05%)	111 (54.95%)	p < 0.001
Skin involvement				
No	362	222 (61.33%)	140 (38.67%)	1.000
Yes	3	2 (66.67%)	1 (33.33%)	1.000
Uterus/ovary involvement				
No	358	220 (61.45%)	138 (38.55%)	1.000
Yes	7	4 (57.14%)	3 (42.86%)	1.000
Breast involvement				
No	343	212 (61.81%)	131 (38.19%)	0.651
Yes	22	12 (54.55%)	10 (45.45%)	0.651
Testis involvement				
No	352	214 (60.80%)	138 (39.20%)	0.377
Yes	13	10 (76.92%)	3 (23.08%)	0.377
CNS involvement				
No	333	208 (62.46%)	125 (37.54%)	0.233
Yes	32	16 (50.00%)	16 (50.00%)	0.233
Thyroid involvement				
No	349	212 (60.74%)	137 (39.26%)	0.377
Yes	16	12 (75.00%)	4 (25.00%)	0.377
Tonsil involvement				
No	346	212 (61.27%)	134 (38.73%)	1.000
Yes	19	12 (63.16%)	7 (36.84%)	1.000
Pancreas involvement				
No	347	223 (64.27%)	124 (35.73%)	p < 0.001
Yes	18	1 (5.56%)	17 (94.44%)	p < 0.001
Liver involvement				
No	340	221 (65.00%)	119 (35.00%)	p < 0.001
Yes	25	3 (12.00%)	22 (88.00%)	p < 0.001
Nasal cavity involvement				
No	340	219 (64.41%)	121 (35.59%)	p < 0.001
Yes	25	5 (20.00%)	20 (80.00%)	p < 0.001
Kidney/adrenal involvement				
No	347	221 (63.69%)	126 (36.31%)	p < 0.001
Yes	18	3 (16.67%)	15 (83.33%)	p < 0.001
Intestine involvement				
No	334	211 (63.17%)	123 (36.83%)	0.033
Yes	31	13 (41.94%)	18 (58.06%)	0.033
Lung involvement				
No	333	212 (63.66%)	121 (36.34%)	0.007
Yes	32	12 (37.50%)	20 (62.50%)	0.007
Stomach involvement				
No	308	182 (59.09%)	126 (40.91%)	0.054
Yes	57	42 (73.68%)	15 (26.32%)	0.054
Mediastinal involvement				
No	360	223 (61.94%)	137 (38.06%)	0.147
Yes	5	1 (20.00%)	4 (80.00%)	0.147
Bone marrow involvement				
No	338	222 (65.68%)	116 (34.32%)	p < 0.001
Yes	27	2 (7.41%)	25 (92.59%)	p < 0.001
Bone involvement				
No	307	216 (70.36%)	91 (29.64%)	p < 0.001
Yes	58	8 (13.79%)	50 (86.21%)	p < 0.001
Therapeutic efficacy				
CR	85	58 (68.24%)	27 (31.76%)	0.208
PD	47	27 (57.45%)	20 (42.55%)	0.208
PR	125	73 (58.40%)	52 (41.60%)	0.208
SD	25	19 (76.00%)	6 (24.00%)	0.208
IPI group				
0–2	228	167 (73.25%)	61 (26.75%)	p < 0.001
3–5	137	57 (41.61%)	80 (58.39%)	p < 0.001
ECOG score				
> = 2	83	39 (46.99%)	44 (53.01%)	0.002
0–1	271	180 (66.42%)	91 (33.58%)	0.002

Multisite ENI was associated with a more adverse baseline clinical profile. Compared with single-site ENI, multisite ENI was more frequently accompanied by age >  = 60 years, Ann Arbor stage III-IV disease, elevated LDH, ECOG performance status >  = 2, and IPI 3–5, whereas ENI burden was not significantly associated with sex or cell-of-origin subtype (Fig. 1A; Table 1). These baseline differences indicate that ENI burden substantially overlaps with established clinical-risk features and should not be interpreted as an isolated disease-intrinsic variable.

Treatment-response patterns were evaluated after the baseline comparison. In the response-evaluable cohort, response distributions differed nominally between patients with and without ENI, with lower complete-response and higher progressive-disease proportions among ENI patients (Fig. 1B). Organ-level response distributions were visualized across involved extranodal sites (Fig. 1C), and organ-response screening suggested nominal associations for CNS and lung involvement (Fig. 1D). Because response was evaluable only in a subset of ENI patients and response availability was incomplete, these therapeutic-efficacy comparisons were interpreted as descriptive available-case analyses rather than adjusted comparative treatment-effect estimates.

### Anatomical distribution and co-involvement patterns of extranodal sites

**Fig. 2:**
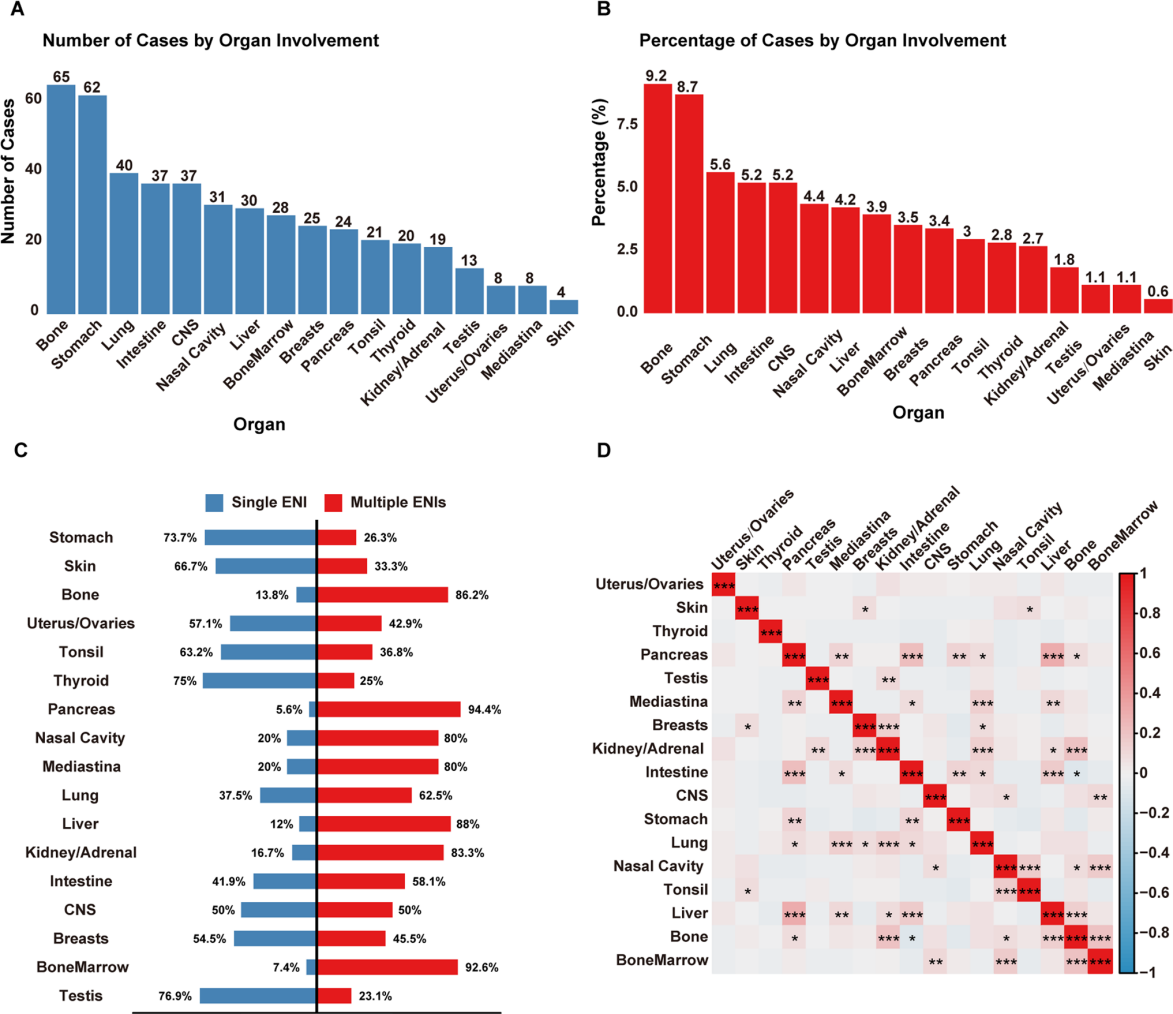
Anatomical distribution and co-involvement of ENI sites in DLBCL. (**A**) Number of patients with involvement of each extranodal organ in the full clinical cohort (n = 710). The most frequent sites were bone (n = 65), stomach (n = 62), lung (n = 40), intestine (n = 37), and CNS (n = 37). (**B**) Percentage of the full cohort with each organ involvement. (**C**) Proportion of single-site and multisite ENI within each organ-defined subgroup. Pancreas, bone marrow, liver, bone, kidney/adrenal gland, nasal cavity, lung, and intestine were enriched in multisite ENI, whereas stomach, thyroid, testis, skin, and tonsil more often appeared as single-site ENI. (**D**) Pairwise correlations among ENI sites; red denotes positive co-involvement and blue denotes negative correlation. Asterisks indicate nominal statistical significance. Organ counts in this figure describe full-cohort anatomical prevalence and may differ from burden-comparison table counts because of available-case denominators and annotation requirements. Counts are not mutually exclusive. Bone marrow, tonsil/Waldeyer’s ring, and mediastinal categories were interpreted according to the boundary-organ rules described in the Methods. CNS, central nervous system; DLBCL, diffuse large B-cell lymphoma; ENI, extranodal involvement

After establishing the clinical correlates of ENI burden, we examined the anatomical distribution of extranodal disease. Organ frequencies across the full clinical cohort are shown as patient counts in Fig. 2A and as percentages of the full cohort in Fig. 2B. The most frequent extranodal sites were bone, stomach, lung, intestine, and CNS. Figure 2 describes full-cohort anatomical prevalence, whereas Table 1 compares organ-defined variables within the available burden-classified ENI subset; differences between figure-level counts and table-level counts therefore reflect analytic denominator and annotation requirements rather than contradictory estimates.

The relationship between organ site and ENI burden was heterogeneous. The proportion of single-site versus multisite ENI within each organ-defined subgroup is displayed in Fig. 2C. Pancreatic, bone marrow, hepatic, skeletal, kidney/adrenal, nasal cavity, pulmonary, and intestinal involvement tended to be enriched in multisite ENI, whereas stomach, thyroid, testis, skin, and tonsillar involvement more often appeared as localized ENI (Fig. 2C; Table 1). These organ-defined patterns were treated as descriptive because several rare sites had small subgroup sizes, unstable denominators, and potential classification overlap with stage or nodal-adjacent anatomy.

Pairwise site-correlation analysis suggested non-random co-involvement patterns (Fig. 2D). The most interpretable pattern was concordance between skeletal and marrow disease, with additional positive associations linking liver and bone, kidney/adrenal gland with intestine and nasal cavity, and several disseminated-site combinations. Conversely, stomach, thyroid, testis, and skin showed fewer positive correlations with other organs, consistent with their more localized presentation in this cohort. These findings describe anatomical co-distribution and do not establish organ-specific biological coupling, because co-involvement may also reflect advanced stage, imaging intensity, referral patterns, and ascertainment bias.

### Survival outcomes and prognostic impact of site-specific ENI

**Fig. 3:**
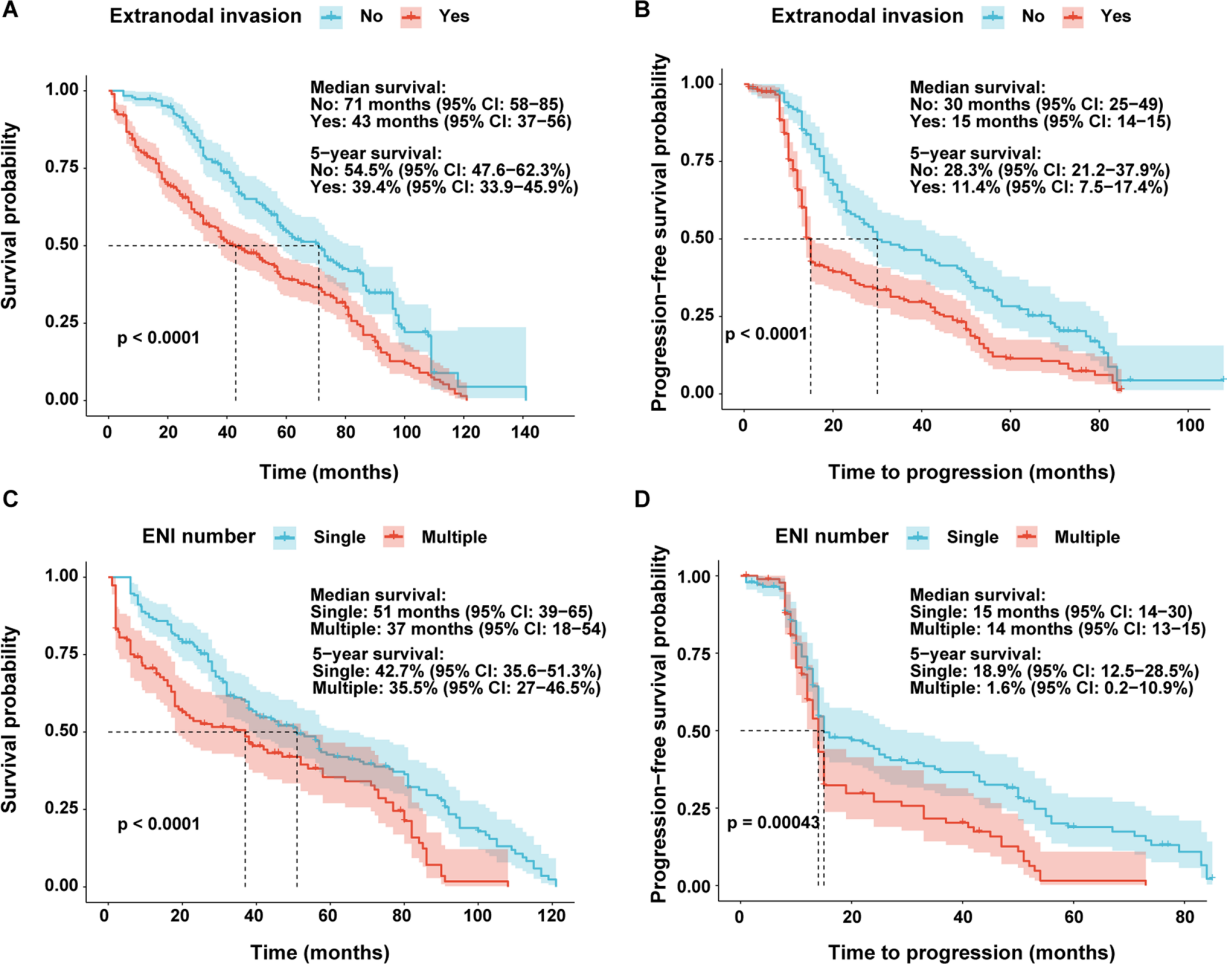
Survival impact of ENI in DLBCL. Kaplan–Meier curves were generated for the full clinical cohort (n = 710) and compared using unadjusted log-rank tests. (**A**) Overall survival according to ENI status: median OS was 71 months in non-ENI patients and 43 months in ENI patients; estimated 5-year OS was 54.5% versus 39.4% (p < 0.0001). (**B**) Progression-free survival according to ENI status: median PFS was 30 months in non-ENI patients and 15 months in ENI patients; estimated 5-year PFS was 28.3% versus 11.4% (p < 0.0001). (**C**) Among ENI patients, single-site ENI was associated with longer unadjusted OS than multisite ENI: median OS 51 versus 37 months; estimated 5-year OS 42.7% versus 35.5% (p < 0.0001). (**D**) PFS among ENI patients stratified by single-site versus multisite ENI: median PFS 15 versus 14 months; estimated 5-year PFS 18.9% versus 1.6% (p = 0.00043). Shaded bands denote 95% confidence intervals. These curves are unadjusted; late 5-year estimates, particularly in multisite ENI, should be interpreted with numbers at risk, censoring, and follow-up maturity. ENI, extranodal involvement; OS, overall survival; PFS, progression-free survival

Survival analyses were performed after the clinical and anatomical descriptions. In the full cohort, patients with ENI had inferior unadjusted OS compared with patients without ENI (median OS, 43 versus 71 months; estimated 5-year OS, 39.4% versus 54.5%; log-rank p < 0.0001; Fig. 3A). ENI was also associated with inferior unadjusted PFS (median PFS, 15 versus 30 months; estimated 5-year PFS, 11.4% versus 28.3%; log-rank p < 0.0001; Fig. 3B). These Kaplan–Meier comparisons were unadjusted and were interpreted together with censoring and number-at-risk patterns because accrual extended to December 2024.

Among patients with ENI, multisite involvement was associated with inferior unadjusted OS and PFS compared with single-site involvement. Median OS was 37 months in multisite ENI and 51 months in single-site ENI, with estimated 5-year OS of 35.5% and 42.7%, respectively (Fig. 3C). PFS separation was more pronounced: median PFS was 14 months in multisite ENI and 15 months in single-site ENI, with estimated 5-year PFS of 1.6% and 18.9%, respectively (Fig. 3D). The very low 5-year PFS estimate in multisite ENI should be interpreted conservatively as a high-risk clinical signal rather than as a precise population estimate, given censoring, event distribution, and available numbers at risk at later time points.

Cox regression analyses were used to distinguish unadjusted prognostic associations from adjusted clinical-risk signals (Table 2). In univariable models, several extranodal sites were associated with inferior survival, including CNS, nasal cavity, kidney/adrenal gland, lung, mediastinum, bone marrow, and bone involvement. In multivariable adjustment models, mediastinal involvement retained adverse PFS and OS signals (PFS: HR 6.22, 95% CI 1.45–26.75, p = 0.014; OS: HR 11.35, 95% CI 2.61–49.45, p = 0.001), whereas CNS involvement retained an adverse OS signal (HR 1.73, 95% CI 1.07–2.80, p = 0.025). Because multiple anatomical sites were screened and several subgroups contained few evaluable patients and events, these site-specific estimates were treated as sparse-data, hypothesis-generating signals rather than definitive independent risk classifiers.

**Table 2:** Cox regression analysis of factors associated with PFS and OS in DLBCL. Hazard ratios are shown with 95% confidence intervals. Unless otherwise specified, hazard ratios compare the higher-risk or presence category with the reference category: age >  = 60 years versus < 60 years; male versus female; elevated LDH versus normal LDH; non-GCB versus GCB; Ann Arbor stage III-IV versus I-II; organ involvement present versus absent; multisite ENI versus single-site ENI; IPI 3–5 versus 0–2; and ECOG performance status >  = 2 versus 0–1. Univariable models were fitted separately for each covariate. Multivariable models were interpreted as adjustment models rather than definitive causal or variable-selection models because of expected collinearity among stage, LDH, ECOG status, IPI, ENI number, and site-specific involvement. Model-specific sample size and event numbers varied according to covariate availability. Rare-site estimates, particularly those with wide confidence intervals, are hypothesis-generating because of sparse events and multiplicity. Abbreviations: CI, confidence interval; CNS, central nervous system; ECOG, Eastern Cooperative Oncology Group; HR, hazard ratio; OS, overall survival; PFS, progression-free survival

Variable	Univ. PFS P	Univ. PFS HR (95% CI)	Univ. OS P	Univ. OS HR (95% CI)	Multiv. PFS P	Multiv. PFS HR (95% CI)	Multiv. OS P	Multiv. OS HR (95% CI)
Age	< 0.001	1.73 (1.39–2.15)	< 0.001	1.55 (1.27–1.88)	0.083	1.34 (0.96–1.87)	0.417	1.14 (0.84–1.54)
Sex	0.365	0.91 (0.73–1.12)	0.937	0.99 (0.82–1.20)				
Elevated serum LDH	< 0.001	2.54 (2.02–3.19)	< 0.001	2.57 (2.09–3.17)	< 0.001	1.92 (1.34–2.75)	< 0.001	1.99 (1.44–2.75)
Subtype	0.982	1.00 (0.79–1.26)	0.619	1.05 (0.85–1.30)				
Ann Arbor stage	< 0.001	2.22 (1.78–2.76)	< 0.001	1.81 (1.48–2.21)	0.002	1.77 (1.24–2.55)	0.050	1.39 (1.00–1.92)
Skin involvement	0.614	1.66 (0.23–11.90)	0.309	2.06 (0.51–8.34)				
Uterus/ovary involvement	0.954	1.02 (0.46–2.31)	0.775	0.89 (0.40–1.99)				
Breast involvement	0.680	1.13 (0.63–2.01)	0.683	1.11 (0.66–1.87)				
Testis involvement	0.616	0.83 (0.41–1.70)	0.360	0.72 (0.35–1.46)				
CNS involvement	< 0.001	2.05 (1.39–3.04)	< 0.001	2.96 (2.04–4.30)	0.551	1.17 (0.70–1.96)	0.025	1.73 (1.07–2.80)
Thyroid involvement	0.551	1.20 (0.66–2.20)	0.330	1.33 (0.75–2.37)				
Tonsil involvement	0.238	1.53 (0.76–3.09)	0.741	1.09 (0.65–1.83)				
Pancreas involvement	0.364	1.36 (0.70–2.65)	0.338	1.38 (0.71–2.69)				
Liver involvement	0.070	1.60 (0.96–2.65)	0.207	1.41 (0.83–2.42)				
Nasal cavity involvement	0.032	1.64 (1.04–2.59)	0.050	1.53 (1.00–2.33)	0.296	1.36 (0.76–2.42)		
Kidney/adrenal involvement	0.028	1.97 (1.08–3.60)	< 0.001	2.65 (1.49–4.72)	0.294	0.68 (0.33–1.40)	0.509	1.27 (0.63–2.54)
Intestine involvement	0.244	1.30 (0.83–2.04)	0.724	1.08 (0.70–1.67)				
Lung involvement	0.037	1.58 (1.03–2.42)	0.002	1.83 (1.24–2.70)	0.521	1.20 (0.68–2.12)	0.325	1.28 (0.78–2.10)
Stomach involvement	0.236	1.24 (0.87–1.78)	0.493	1.13 (0.79–1.61)				
Mediastinal involvement	0.047	3.18 (1.02–9.97)	< 0.001	8.82 (2.79–27.88)	0.014	6.22 (1.45–26.75)	0.001	11.35 (2.61–49.45)
Bone marrow involvement	< 0.001	2.33 (1.48–3.66)	< 0.001	2.90 (1.85–4.53)	0.955	0.98 (0.56–1.72)	0.147	1.48 (0.87–2.50)
Bone involvement	< 0.001	2.77 (1.94–3.95)	< 0.001	2.30 (1.63–3.25)	0.148	1.43 (0.88–2.33)	0.190	1.35 (0.86–2.12)
ENI number	< 0.001	1.72 (1.27–2.33)	< 0.001	1.77 (1.34–2.34)	0.705	0.92 (0.60–1.41)	0.948	0.99 (0.69–1.42)
IPI group	< 0.001	1.65 (1.31–2.08)	< 0.001	1.58 (1.28–1.94)	0.880	0.97 (0.68–1.38)	0.838	0.97 (0.69–1.35)
ECOG score	< 0.001	2.07 (1.60–2.68)	< 0.001	2.72 (2.13–3.46)	0.062	1.38 (0.98–1.94)	0.002	1.69 (1.22–2.33)

Established clinical risk factors remained important in the adjusted models. Elevated LDH and Ann Arbor stage III-IV were associated with inferior PFS, and ECOG performance status >  = 2 contributed to OS risk (Table 2). In contrast, ENI number and most individual anatomical sites lost statistical significance after adjustment. This pattern supports the main adjusted interpretation: much of the adverse prognostic association of ENI burden appears to be mediated or confounded by systemic disease burden, baseline host fitness, and treatment feasibility, although selected anatomical sites warrant validation in larger cohorts with harmonized site definitions and mature follow-up.

### Exploratory genomic landscape and ENI-associated mutational patterns

**Fig. 4:**
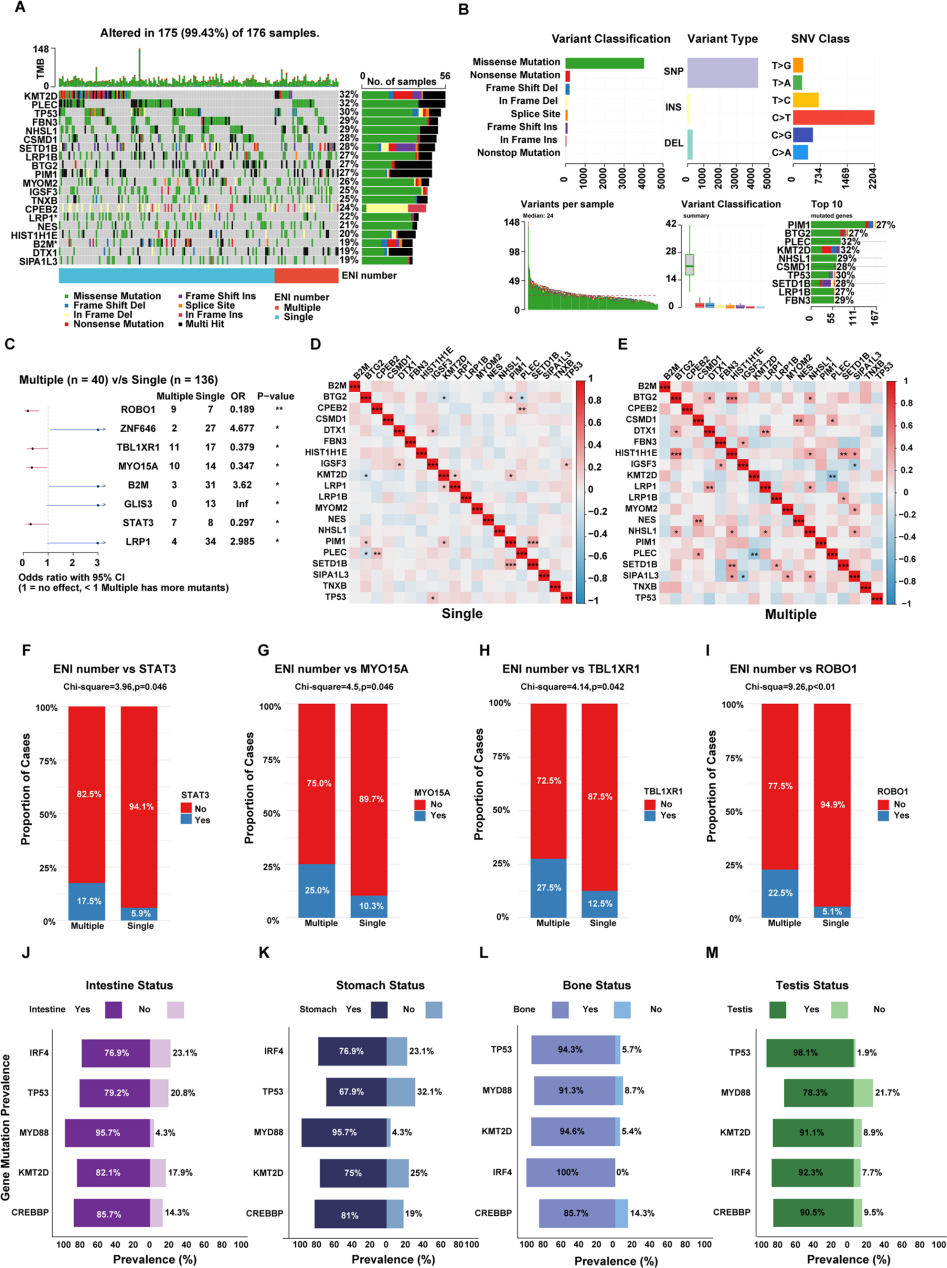
Exploratory genomic landscape and ENI-associated alteration patterns in DLBCL. Targeted next-generation sequencing was performed in 176 tumors with available diagnostic material. (**A**) Oncoprint of detected alterations, annotated by ENI status. (**B**) Variant summary, including variant classification, variant type, SNV class, variant burden per sample, and the most frequently altered genes. (**C**) Differential alteration analysis comparing multisite versus single-site ENI identified candidate alteration patterns involving B2M, STAT3, MYO15A, TBL1XR1, and ROBO1. (**D**-**E**) Pairwise co-occurrence and mutual-exclusivity matrices in single-site and multisite ENI. (**F**-**I**) Representative alterations associated with ENI number. (**J**-**M**) Site-specific alteration prevalence according to intestine, stomach, bone, and testis involvement. Because the assay used a targeted panel, matched-normal sequencing was incomplete, tissue availability was non-random, and multiple genes were tested, these findings are exploratory tumor-panel alteration signals and should not be interpreted as validated ENI-defining genomic events. DLBCL, diffuse large B-cell lymphoma; ENI, extranodal involvement; OR, odds ratio; SNV, single-nucleotide variant

Targeted sequencing was performed in 176 tumors with available diagnostic material, and at least one detected genetic alteration was identified in 175 tumors (99.43%). The overall alteration landscape and ENI annotation are shown in the oncoprint (Fig. 4A), and the variant summary, including variant classification, variant type, SNV class, variant burden, and the most frequently altered genes, is shown in Fig. 4B. The most frequently altered genes included KMT2D and PLEC (32% each), TP53 (30%), NHSL1 and FBN3 (29% each), CSMD1 and SETD1B (28% each), and LRP1B, BTG2, and PIM1 (27% each). Because matched-normal sequencing was not uniformly available, these results were reported as tumor-panel alteration patterns rather than definitive somatic driver events.

Exploratory comparison of single-site and multisite ENI identified differential alteration signals across selected genes, including B2M, STAT3, MYO15A, TBL1XR1, and ROBO1 (Fig. 4C). In this cohort, B2M alterations were more frequently observed in single-site ENI, whereas STAT3-, MYO15A-, TBL1XR1-, and ROBO1-associated alterations were relatively enriched in multisite ENI. Gene-level comparisons were interpreted in the context of multiple testing, panel design, tumor-only sequencing, mutation burden, biopsy-site selection, and tissue availability; therefore, these findings should be regarded as exploratory candidate signals rather than validated ENI-defining genomic events.

Pairwise mutation analyses showed recurrent co-occurrence and mutual-exclusivity patterns in both ENI subgroups (Fig. 4D, E). In single-site ENI, co-occurrence was observed for B2M-BTG2, BTG2-PIM1, CPEB2-KMT2D, FBN3-IGSF3, and SETD1B-SIPA1L3, whereas TP53 showed mutual exclusivity with SIPA1L3 and SETD1B (Fig. 4D). Similar patterns appeared in multisite ENI, including B2M-BTG2, CPEB2-KMT2D, FBN3-IGSF3, and TP53-SIPA1L3 (Fig. 4E). These relationships may be influenced by mutation burden, panel composition, tumor purity, and sparse subgroups and were not interpreted as pathway-level dependencies without independent confirmation.

Additional alteration patterns were examined according to ENI number and selected anatomical sites. Alterations associated with ENI burden are displayed in Fig. 4F-I, and representative site-specific genomic patterns are summarized in Fig. 4J-M. Site-specific alteration prevalence for intestine, stomach, bone, and testis involvement showed observed differences across selected DLBCL-related genes, including IRF4, TP53, MYD88, KMT2D, and CREBBP. These findings raise the possibility that anatomical context and molecular profile may be linked in some forms of extranodal DLBCL, but the current molecular sample size is not adequate for site-specific molecular taxonomy.

### Exploratory transcriptomic and immune microenvironmental features of ENI

**Fig. 5:**
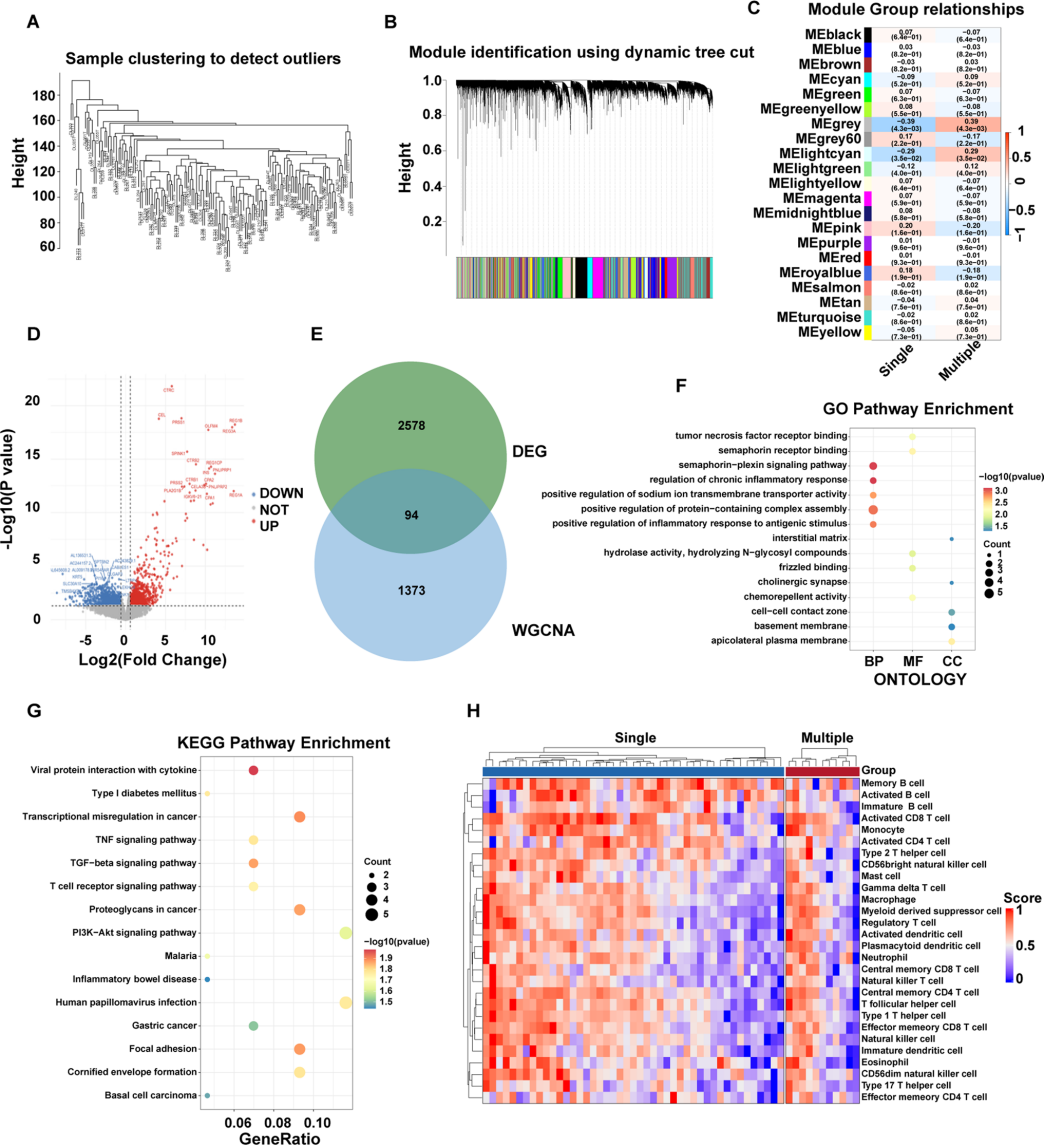
Exploratory transcriptomic and immune-signature analysis of ENI. RNA-seq analysis was performed in 107 quality-controlled tumor transcriptomes. (**A**) Hierarchical clustering was used to assess sample-level structure and potential outliers. (**B**) WGCNA module identification using dynamic tree cutting. (**C**) Module-trait correlations showed association of the grey60 module with multisite ENI and the lightcyan module with single-site ENI. (**D**) Volcano plot of differential expression between ENI subgroups. (**E**) Overlap between differentially expressed genes and phenotype-associated WGCNA module genes yielded 94 prioritized genes; 2,578 genes were unique to the differential-expression set and 1,373 were unique to the WGCNA-associated set. (**F**) GO enrichment analysis of overlapping genes. (**G**) KEGG pathway enrichment analysis, including TNF, TGF-beta, T-cell receptor, and PI3K-Akt signaling. (**H**) Heatmap of immune-cell signature scores estimated by ssGSEA. Because these signatures were inferred from bulk RNA-seq, they represent relative transcriptional patterns rather than direct measurements of immune-cell abundance, spatial localization, or functional state. Orthogonal validation by immunohistochemistry, flow cytometry, single-cell sequencing, or spatial profiling is required. ENI, extranodal involvement; GO, Gene Ontology; KEGG, Kyoto Encyclopedia of Genes and Genomes; ssGSEA, single-sample gene-set enrichment analysis; WGCNA, weighted gene co-expression network analysis

RNA-seq and immune-signature analyses were performed in 107 tumor samples that passed transcriptomic quality control. Hierarchical clustering was first used to examine sample-level structure and potential outliers (Fig. 5A). WGCNA then defined co-expression modules using dynamic tree cutting (Fig. 5B), and module-trait correlation analysis linked the grey60 module with multisite ENI and the lightcyan module with single-site ENI (Fig. 5C). Because the profiled specimen was selected according to tissue availability and diagnostic adequacy, these transcriptomic results were interpreted as exploratory tissue-level signals, particularly in multisite disease where a single sampled lesion may not represent all involved organs.

Differential expression analysis identified genes that differed between ENI subgroups (Fig. 5D). Intersecting differentially expressed genes with phenotype-associated WGCNA module genes yielded 94 overlapping genes, whereas 2,578 genes were unique to the differential-expression set and 1,373 were unique to the WGCNA-associated set (Fig. 5E). The 94 overlapping genes were prioritized because they were supported by two complementary analytical strategies; however, this overlap was used as a filtering strategy rather than as a definitive core-gene definition.

Functional enrichment of the 94 overlapping genes highlighted immune signaling, inflammatory regulation, and extracellular-matrix organization. GO enrichment included TNF receptor binding, semaphorin-plexin signaling, regulation of chronic inflammatory response, basement membrane structure, interstitial matrix organization, and cell–cell junction organization (Fig. 5F). KEGG enrichment included TNF, TGF-beta, T-cell receptor, and PI3K-Akt signaling, together with focal adhesion and proteoglycans in cancer (Fig. 5G). These pathway-level results were interpreted as exploratory and were emphasized only when supported by FDR-controlled enrichment or concordant biological context.

Bulk RNA-seq further suggested differential immune and stromal transcriptional states across ENI patterns. Single-site ENI showed higher memory B-cell and activated T-cell signatures, whereas multisite ENI showed higher regulatory T-cell and myeloid-derived suppressor-cell signatures (Fig. 5H). Because ssGSEA estimates derive from bulk tissue transcriptomes, they cannot directly establish immune-cell abundance, spatial localization, or functional immune suppression. Concordant trends across ssGSEA and deconvolution methods such as CIBERSORTx or xCell were therefore used only as consistency checks, and the immune-signature findings remain exploratory hypotheses requiring validation by immunohistochemistry, flow cytometry, single-cell sequencing, or spatial profiling.

## Discussion

This study examined ENI in newly diagnosed DLBCL by integrating clinical, anatomical, targeted genomic, and transcriptomic information. The central finding is that ENI should not be treated as a single uniform entity. ENI was associated with inferior outcome in unadjusted analyses, but multisite ENI was also tightly linked to age, stage, LDH, performance status, and IPI. After adjustment for established clinical risk factors, the prognostic effect of ENI burden was attenuated. These results support a burden- and site-aware clinical view of ENI while cautioning against interpreting multisite ENI as an independent biological category or stand-alone prognostic determinant without further validation. This interpretation is consistent with prior clinical studies showing that the prognostic meaning of extranodal disease varies according to the number and site of involvement rather than behaving as a uniformly adverse binary feature [31–35]. It is also aligned with contemporary DLBCL pathology and extranodal DLBCL reviews, which emphasize diagnostic and biological heterogeneity across DLBCL presentations [36, 37], and with recent multicenter evidence that extranodal DLBCL contains clinically and molecularly diverse subsets [38].

### Exploratory genomic and immune-transcriptomic interpretation

The genomic analysis identified exploratory alteration patterns that may accompany ENI burden rather than a single defining genomic event. Differential alteration patterns involving B2M, STAT3, MYO15A, TBL1XR1, and ROBO1 were observed across ENI subgroups, although the directionality and biological implications of these signals require cautious interpretation. Alterations affecting antigen presentation and immune recognition, including B2M and HLA-related mechanisms, have been implicated in DLBCL immune escape, whereas broader genomic studies have shown that DLBCL molecular structure is heterogeneous rather than defined by a single alteration [4–8, 11, 12]. Because the sequencing strategy relied on a targeted panel without uniform matched-normal sequencing or orthogonal protein-level validation, the present data are insufficient to infer causal mechanisms of extranodal dissemination. Instead, these findings should be viewed as candidate genomic correlates of ENI patterns that require validation before being incorporated into molecular taxonomy.

The transcriptomic findings point in a related but still exploratory direction. Single-site ENI retained relatively stronger adaptive immune signatures, whereas multisite ENI showed transcriptional programs compatible with relatively stronger regulatory T-cell and myeloid-derived suppressor-cell states. These observations support immune-transcriptional hypotheses rather than definitive mechanistic conclusions. Enrichment of TNF, TGF-beta, T-cell receptor, extracellular-matrix, and PI3K-Akt signaling pathways further suggests that extranodal patterns may be accompanied by different immune-microenvironmental signals, consistent with prior work showing that DLBCL tumor-microenvironment programs and immune-cell signatures are clinically and biologically informative [13, 14]. Nevertheless, bulk RNA-seq remains a population-level readout and cannot resolve cellular localization, direct tumor-immune interaction, or functional immune suppression; deconvolution and signature-based approaches should therefore be regarded as indirect estimates requiring orthogonal validation [26–28]. Therefore, these findings should be interpreted as hypothesis-generating observations that require orthogonal validation using immunohistochemistry, flow cytometry, single-cell sequencing, or spatial transcriptomic approaches.

### Integrative interpretation and translational significance

The value of the study lies less in any single site-specific or gene-specific observation than in the integrative conceptual framework generated by combining clinical burden, anatomical distribution, genomic screening, and transcriptomic immune context. The findings suggest that ENI in DLBCL is not adequately captured by a binary adverse-risk label. Instead, extranodal disease appears to contain layered clinical structure involving burden-dependent dissemination patterns and organ-specific distribution, with exploratory genomic and immune-transcriptional correlates that require independent validation. This framework also aligns with contemporary DLBCL literature highlighting the relevance of immune escape, antigen-presentation defects, and microenvironmental programs to biological heterogeneity and outcome [11–14].

From a translational perspective, the present data do not justify immediate ENI-driven therapeutic stratification. Rather, they support more granular annotation of extranodal disease in future DLBCL studies and provide a rationale for prospective multicenter investigations integrating standardized staging, treatment-intensity metrics, matched-normal sequencing, and spatial immune profiling [16, 21, 37, 38]. Such approaches may determine whether ENI-associated immune-molecular states contribute additional prognostic or therapeutic information beyond existing clinical risk systems such as the IPI and NCCN-IPI [9, 10].

### Limitations and safeguards for interpretation

Several limitations should be considered when interpreting these findings. The retrospective design creates unavoidable risks of selection bias, missing data, and non-random treatment allocation. Available-case denominators differ across clinical, anatomical, response, survival, genomic, and transcriptomic analyses; therefore, figure-level and table-level counts should not be interpreted as interchangeable. ENI burden is also clinically and statistically intertwined with advanced stage, LDH elevation, ECOG performance status, IPI, and treatment feasibility, which are core components of established DLBCL risk assessment [9, 10]. In addition, rare anatomical sites, including mediastinal involvement, produced wide confidence intervals and unstable estimates, and multiple exploratory comparisons were interpreted with attention to false-discovery control [29]. The molecular analyses were limited by tissue availability, targeted-panel design, incomplete matched-normal sequencing, modest RNA-seq sample size, single-lesion sampling in multisite disease, and immune inference from bulk RNA rather than direct cellular or spatial measurement [21, 26–28]. Detailed morphologic subtype annotation was also not uniformly archived in the retrospective records, so a robust morphology-genomics correlation analysis could not be performed across the full cohort.

### Future directions

Future studies should prospectively capture ENI burden using harmonized site definitions, record treatment regimen and dose intensity in a standardized manner, preserve numbers at risk for mature survival estimates, and incorporate standardized pathology review with prospective recording of morphologic subtype. Molecular validation should include matched-normal sequencing, systematic sampling or spatial profiling of representative lesions, and orthogonal immune assays such as immunohistochemistry, flow cytometry, single-cell sequencing, or spatial transcriptomics. Such work will be needed to determine whether ENI-associated anatomical and immune-molecular patterns provide information beyond established clinical risk models and whether they can eventually inform therapeutic stratification.

## Conclusions

In summary, ENI in DLBCL should not be regarded as a uniform binary descriptor. In this retrospective cohort, extranodal burden and anatomical distribution identified clinically heterogeneous patterns associated with adverse baseline features and inferior unadjusted outcomes, extending prior reports that the number and site of ENI carry non-equivalent clinical meaning [31–35]. This conclusion should be interpreted in the context of contemporary DLBCL pathology and extranodal DLBCL literature, which emphasize diagnostic heterogeneity, site-specific biological features, and variable risks of dissemination or central nervous system relapse across extranodal presentations [36, 37]. It is also consistent with recent multicenter evidence that extranodal DLBCL contains clinically and molecularly diverse subsets rather than a single homogeneous category [38]. However, the independent prognostic contribution of ENI burden was attenuated after adjustment for established clinical-risk factors, indicating substantial overlap with systemic disease burden, host fitness, and treatment feasibility. Exploratory targeted sequencing and transcriptomic analyses suggested candidate genomic and immune-transcriptional correlates of ENI patterns that are biologically compatible with published work on immune escape and microenvironmental heterogeneity in DLBCL [11–14], but these findings do not establish a validated biological subtype or treatment-selection framework.

This interpretation remains constrained by the retrospective single-institution design, limited maturity of long-term survival estimates, sparse site-defined subgroups, variable-specific available-case denominators, treatment-intensity heterogeneity, targeted-panel sequencing, incomplete matched-normal confirmation, modest RNA-seq sample size, lack of orthogonal immune validation, and non-uniform archival recording of detailed morphologic subtypes. Prospective multicenter studies integrating harmonized staging, treatment-intensity annotation, standardized pathology review, matched-normal sequencing, spatial immune profiling, and mature follow-up will be necessary before ENI-informed biological stratification can guide clinical decision-making.

## Supplementary Information

Below is the link to the electronic supplementary material.

Supplementary file1 (TIF 16574 KB) Study workflow and analytical framework for extranodal involvement (ENI) heterogeneity in diffuse large B-cell lymphoma (DLBCL). The retrospective clinical cohort included 710 adults with newly diagnosed DLBCL treated with first-line immunochemotherapy. ENI was evaluated at diagnosis and classified as absent, single-site ENI (n = 223), or multisite ENI (n = 141) within the adjudicated ENI cohort (n = 364). Analyses were organized into clinical/anatomical, survival, targeted sequencing, and RNA-seq layers. Clinical and anatomical analyses used available-case denominators; survival analyses separated unadjusted Kaplan–Meier comparisons from multivariable Cox adjustment models; molecular analyses were considered exploratory owing to tissue availability, sample size, incomplete matched-normal sequencing, sparse subgroups, and bulk RNA-seq inference. The workflow highlights that ENI should be interpreted as a burden- and site-aware phenotype rather than as a uniform binary prognostic category

Supplementary file2 (DOCX 12 KB)

## Data Availability

De-identified clinical data may be made available by the corresponding author upon reasonable request, subject to institutional ethics approval, consent restrictions, and applicable data-protection regulations. Sequencing-derived data and processed expression matrices will be deposited in an appropriate controlled-access repository when permitted by consent and local regulation. Analysis scripts, figure-generation code, and key parameter files will be shared through a public or controlled repository before publication or during peer review, with protected patient-level information removed. If public release is restricted, a version-controlled code archive will be made available to editors and reviewers upon request.

## References

[1] Swerdlow SH, Campo E, Pileri SA, Harris NL, Stein H, Siebert R, et al. The 2016 revision of the World Health Organization classification of lymphoid neoplasms. Blood. 2016;127:2375–90. 10.1182/blood-2016-01-643569. 26980727 10.1182/blood-2016-01-643569 PMC4874220

[2] Sehn LH, Salles G. Diffuse large B-cell lymphoma. N Engl J Med. 2021;384:842–58. 10.1056/NEJMra2027612. 33657296 10.1056/NEJMra2027612 PMC8377611

[3] Alizadeh AA, Eisen MB, Davis RE, Ma C, Lossos IS, Rosenwald A, et al. Distinct types of diffuse large B-cell lymphoma identified by gene expression profiling. Nature. 2000;403:503–11. 10.1038/35000501. 10676951 10.1038/35000501

[4] Schmitz R, Wright GW, Huang DW, Johnson CA, Phelan JD, Wang JQ, et al. Genetics and pathogenesis of diffuse large B-cell lymphoma. N Engl J Med. 2018;378:1396–407. 10.1056/NEJMoa1801445. 29641966 10.1056/NEJMoa1801445 PMC6010183

[5] Chapuy B, Stewart C, Dunford AJ, Kim J, Kamburov A, Redd RA, et al. Molecular subtypes of diffuse large B cell lymphoma are associated with distinct pathogenic mechanisms and outcomes. Nat Med. 2018;24:679–90. 10.1038/s41591-018-0016-8. 29713087 10.1038/s41591-018-0016-8 PMC6613387

[6] Reddy A, Zhang J, Davis NS, Moffitt AB, Love CL, Waldrop A, et al. Genetic and functional drivers of diffuse large B cell lymphoma. Cell. 2017;171:481-494.e15. 10.1016/j.cell.2017.09.027. 28985567 10.1016/j.cell.2017.09.027 PMC5659841

[7] Wright GW, Huang DW, Phelan JD, Coulibaly ZA, Roulland S, Young RM, et al. A probabilistic classification tool for genetic subtypes of diffuse large B cell lymphoma with therapeutic implications. Cancer Cell. 2020;37:551-568.e14. 10.1016/j.ccell.2020.03.015. 32289277 10.1016/j.ccell.2020.03.015 PMC8459709

[8] Pasqualucci L, Dalla-Favera R. Genetics of diffuse large B-cell lymphoma. Blood. 2018;131:2307–19. 10.1182/blood-2017-11-764332. 29666115 10.1182/blood-2017-11-764332 PMC5969374

[9] The International Non-Hodgkin’s Lymphoma Prognostic Factors Project. A predictive model for aggressive non-Hodgkin’s lymphoma. N Engl J Med. 1993;329:987–994. 10.1056/NEJM199309303291402. 10.1056/NEJM199309303291402 8141877

[10] Zhou Z, Sehn LH, Rademaker AW, Gordon LI, Lacasce AS, Crosby-Thompson A, et al. An enhanced International Prognostic Index for patients with diffuse large B-cell lymphoma treated in the rituximab era. Blood. 2014;123:837–42. 10.1182/blood-2013-09-524108. 24264230 10.1182/blood-2013-09-524108 PMC5527396

[11] Challa-Malladi M, Lieu YK, Califano O, Holmes A, Bhagat G, Murty VV, et al. Combined genetic inactivation of β2-microglobulin and CD58 reveals frequent escape from immune recognition in diffuse large B-cell lymphoma. Cancer Cell. 2011;20:728–40. 10.1016/j.ccr.2011.11.006. 22137796 10.1016/j.ccr.2011.11.006 PMC3660995

[12] Fangazio M, Ladewig E, Gomez K, Garcia-Ibanez L, Kumar R, Teruya-Feldstein J, et al. Genetic mechanisms of HLA-I loss and immune escape in diffuse large B cell lymphoma. Proc Natl Acad Sci U S A. 2021;118:e2104504118. 10.1073/pnas.2104504118. 34050029 10.1073/pnas.2104504118 PMC8179151

[13] Kotlov N, Bagaev A, Revuelta MV, Phillip JM, Cacciapuoti MT, Antysheva Z, et al. Clinical and biological subtypes of B-cell lymphoma revealed by microenvironmental signatures. Cancer Discov. 2021;11:1468–89. 10.1158/2159-8290.CD-20-0839. 33541860 10.1158/2159-8290.CD-20-0839 PMC8178179

[14] Yu H, Fu D, Xu PP, Cheng S, Wang L, Zhang YZ, et al. Implication of immune cell signature of tumor microenvironment in diffuse large B-cell lymphoma. Hematol Oncol. 2021;39:616–24. 10.1002/hon.2905. 34331367 10.1002/hon.2905

[15] Coiffier B, Lepage E, Brière J, Herbrecht R, Tilly H, Bouabdallah R, et al. CHOP chemotherapy plus rituximab compared with CHOP alone in elderly patients with diffuse large-B-cell lymphoma. N Engl J Med. 2002;346:235–42. 10.1056/NEJMoa011795. 11807147 10.1056/NEJMoa011795

[16] Cheson BD, Fisher RI, Barrington SF, Cavalli F, Schwartz LH, Zucca E, et al. Recommendations for initial evaluation, staging, and response assessment of Hodgkin and non-Hodgkin lymphoma: the Lugano classification. J Clin Oncol. 2014;32:3059–68. 10.1200/JCO.2013.54.8800. 25113753 10.1200/JCO.2013.54.8800 PMC4979083

[17] Andrews S FastQC: a quality control tool for high throughput sequence data. Babraham Bioinformatics. 2010. Available from: https://www.bioinformatics.babraham.ac.uk/projects/fastqc/.

[18] Bolger AM, Lohse M, Usadel B. Trimmomatic: a flexible trimmer for Illumina sequence data. Bioinformatics. 2014;30:2114–20. 10.1093/bioinformatics/btu170. 24695404 10.1093/bioinformatics/btu170 PMC4103590

[19] Li H, Durbin R. Fast and accurate short read alignment with Burrows–Wheeler transform. Bioinformatics. 2009;25:1754–60. 10.1093/bioinformatics/btp324. 19451168 10.1093/bioinformatics/btp324 PMC2705234

[20] Danecek P, Bonfield JK, Liddle J, Marshall J, Ohan V, Pollard MO, et al. Twelve years of SAMtools and BCFtools. Gigascience. 2021;10:giab008. 10.1093/gigascience/giab008. 33590861 10.1093/gigascience/giab008 PMC7931819

[21] Van der Auwera GA, O’Connor BD. Genomics in the cloud: using Docker, GATK, and WDL in Terra. Sebastopol, CA: O’Reilly Media; 2020.

[22] McLaren W, Gil L, Hunt SE, Riat HS, Ritchie GRS, Thormann A, et al. The Ensembl variant effect predictor. Genome Biol. 2016;17:122. 10.1186/s13059-016-0974-4. 27268795 10.1186/s13059-016-0974-4 PMC4893825

[23] Ritchie ME, Phipson B, Wu D, Hu Y, Law CW, Shi W, et al. Limma powers differential expression analyses for RNA-sequencing and microarray studies. Nucleic Acids Res. 2015;43:e47. 10.1093/nar/gkv007. 25605792 10.1093/nar/gkv007 PMC4402510

[24] Langfelder P, Horvath S. WGCNA: an R package for weighted correlation network analysis. BMC Bioinformatics. 2008;9:559. 10.1186/1471-2105-9-559. 19114008 10.1186/1471-2105-9-559 PMC2631488

[25] Yu G, Wang LG, Han Y, He QY. ClusterProfiler: an R package for comparing biological themes among gene clusters. OMICS. 2012;16:284–7. 10.1089/omi.2011.0118. 22455463 10.1089/omi.2011.0118 PMC3339379

[26] Hänzelmann S, Castelo R, Guinney J. GSVA: gene set variation analysis for microarray and RNA-seq data. BMC Bioinformatics. 2013;14:7. 10.1186/1471-2105-14-7. 23323831 10.1186/1471-2105-14-7 PMC3618321

[27] Newman AM, Steen CB, Liu CL, Gentles AJ, Chaudhuri AA, Scherer F, et al. Determining cell type abundance and expression from bulk tissues with digital cytometry. Nat Biotechnol. 2019;37:773–82. 10.1038/s41587-019-0114-2. 31061481 10.1038/s41587-019-0114-2 PMC6610714

[28] Aran D, Hu Z, Butte AJ. XCell: digitally portraying the tissue cellular heterogeneity landscape. Genome Biol. 2017;18:220. 10.1186/s13059-017-1349-1. 29141660 10.1186/s13059-017-1349-1 PMC5688663

[29] Benjamini Y, Hochberg Y. Controlling the false discovery rate: a practical and powerful approach to multiple testing. J R Stat Soc Series B Stat Methodol. 1995;57:289–300. 10.1111/j.2517-6161.1995.tb02031.x.

[30] Mayakonda A, Lin DC, Assenov Y, Plass C, Koeffler HP. Maftools: efficient and comprehensive analysis of somatic variants in cancer. Genome Res. 2018;28:1747–56. 10.1101/gr.239244.118. 30341162 10.1101/gr.239244.118 PMC6211645

[31] Castillo JJ, Winer ES, Olszewski AJ. Sites of extranodal involvement are prognostic in patients with diffuse large B-cell lymphoma in the rituximab era: an analysis of the Surveillance, Epidemiology and End Results database. Am J Hematol. 2014;89:310–4. 10.1002/ajh.23638. 24273125 10.1002/ajh.23638

[32] Lu CS, Chen JH, Huang TC, Wu YY, Chang PY, Dai MS, et al. Diffuse large B-cell lymphoma: sites of extranodal involvement are a stronger prognostic indicator than number of extranodal sites in the rituximab era. Leuk Lymphoma. 2015;56:2047–55. 10.3109/10428194.2014.982636. 25382616 10.3109/10428194.2014.982636

[33] Bobillo S, Joffe E, Lavery JA, Sermer D, Ghione P, Noy A, et al. Clinical characteristics and outcomes of extranodal stage I diffuse large B-cell lymphoma in the rituximab era. Blood. 2021;137:39–48. 10.1182/blood.2020005112. 32730585 10.1182/blood.2020005112 PMC8555387

[34] Yoo C, Kim S, Sohn BS, Kim JE, Yoon DH, Huh J, et al. Modified number of extranodal involved sites as a prognosticator in R-CHOP-treated patients with disseminated diffuse large B-cell lymphoma. Korean J Intern Med. 2010;25:301–8. 10.3904/kjim.2010.25.3.301. 20830228 10.3904/kjim.2010.25.3.301 PMC2932944

[35] Oiwa K, Fujita K, Lee S, Morishita T, Tsujikawa T, Negoro E, et al. Prognostic value of metabolic tumor volume of extranodal involvement in diffuse large B cell lymphoma. Ann Hematol. 2023;102:1141–8. 10.1007/s00277-023-05165-x. 36951966 10.1007/s00277-023-05165-x PMC10102098

[36] Li S, Young KH, Medeiros LJ. Diffuse large B-cell lymphoma. Pathology. 2018;50:74–87. 10.1016/j.pathol.2017.09.006. 29167021 10.1016/j.pathol.2017.09.006

[37] Ollila TA, Olszewski AJ. Extranodal diffuse large B cell lymphoma: molecular features, prognosis, and risk of central nervous system recurrence. Curr Treat Options Oncol. 2018;19:38. 10.1007/s11864-018-0555-8. 29931605 10.1007/s11864-018-0555-8 PMC6294323

[38] Chen SY, Xu PP, Feng R, Cui GH, Wang L, Cheng S, et al. Extranodal diffuse large B-cell lymphoma: clinical and molecular insights with survival outcomes from the multicenter EXPECT study. Cancer Commun. 2025;45:919–35. 10.1002/cac2.70033. 10.1002/cac2.70033 PMC12365540 40344323

