# Supplementary material for: Extranodal involvement defines distinct immune-molecular phenotypes and clinical outcomes in diffuse large b-cell lymphoma
Source: Clin Exp Med. 2026 Jul 26;26(1):293. doi: 10.1007/s10238-026-02268-3 (PMC13451387; doi:10.1007/s10238-026-02268-3)
Supplement: Supplementary file 1 [file 10238_2026_2268_MOESM2_ESM.docx]

Supplementary Table S2. Research resources, RRIDs, and traceability information

| **Resource** | **Role in this study** | **Version / release reported in manuscript** | **RRID / identifier status** | **Manuscript-supported source** |
| --- | --- | --- | --- | --- |
| Illumina sequencing platform | Paired-end sequencing platform | Model not specified in manuscript | RRID not assigned because exact instrument model was not available in the manuscript records | Methods Section 2.4 |
| FastQC | Raw-read quality assessment | Not specified in manuscript | RRID:SCR_014583 | Andrews S. FastQC: a quality control tool for high throughput sequence data. 2010. |
| Trimmomatic | Read trimming | Not specified in manuscript | RRID:SCR_011848 | Bolger AM, Lohse M, Usadel B. Bioinformatics. 2014;30:2114-2120. |
| BWA-MEM | Alignment to hg19/GRCh37 | Not specified in manuscript | RRID:SCR_010910 | Li H, Durbin R. Bioinformatics. 2009;25:1754-1760. |
| SAMtools and related tools | Alignment-file processing | Not specified in manuscript | RRID:SCR_002105 | Danecek P, et al. GigaScience. 2021;10:giab008. |
| GATK best-practice principles | Variant-calling workflow framework | Not specified in manuscript | RRID:SCR_001876 | Van der Auwera GA, O'Connor BD. Genomics in the Cloud. 2020. |
| Ensembl Variant Effect Predictor | Variant annotation | Not specified in manuscript | RRID:SCR_007931 | McLaren W, et al. Genome Biol. 2016;17:122. |
| IBM SPSS Statistics | Clinical and survival analyses | 26.0 | RRID:SCR_016479 | Methods Section 2.7 |
| R Project for Statistical Computing | Clinical, transcriptomic, and bioinformatic analyses | 4.3.3 | RRID:SCR_001905 | Methods Section 2.7 |
| limma | Differential expression analysis | Package version not specified in manuscript | RRID:SCR_010943 | Ritchie ME, et al. Nucleic Acids Res. 2015;43:e47. |
| WGCNA | Co-expression network analysis | Package version not specified in manuscript | RRID:SCR_003302 | Langfelder P, Horvath S. BMC Bioinformatics. 2008;9:559. |
| clusterProfiler | Functional enrichment analysis | Package version not specified in manuscript | RRID:SCR_016884 | Yu G, Wang LG, Han Y, He QY. OMICS. 2012;16:284-287. |
| GSVA / ssGSEA framework | Immune-signature estimation | Package version not specified in manuscript | RRID:SCR_021058 | Hanzelmann S, Castelo R, Guinney J. BMC Bioinformatics. 2013;14:7. |
| maftools | Somatic-variant analysis | Package version not specified in manuscript | RRID not confirmed from available verification records; canonical citation retained | Mayakonda A, et al. Genome Res. 2018;28:1747-1756. |
| CIBERSORTx | Deconvolution cross-check | Not specified in manuscript | RRID not confirmed for CIBERSORTx in available verification records; canonical citation retained | Newman AM, et al. Nat Biotechnol. 2019;37:773-782. |
| xCell | Deconvolution cross-check | Not specified in manuscript | RRID not confirmed from available verification records; canonical citation retained | Aran D, Hu Z, Butte AJ. Genome Biol. 2017;18:220. |

Note: This table is restricted to resources explicitly cited or described in the manuscript. RRIDs are provided only where a resource-level identifier could be verified with sufficient confidence from SciCrunch/RRID resolver records. For resources lacking exact model or package-version information in the study records, or for resources without a confirmed resource-level RRID during verification, the table preserves the manuscript-supported information and marks the missing element transparently rather than assigning an unverified identifier.
